# Humoral Response to SARS-Cov-2 Vaccination in Liver Transplant Recipients–A Single-Center Experience

**DOI:** 10.3390/vaccines9070738

**Published:** 2021-07-04

**Authors:** Jassin Rashidi-Alavijeh, Alexandra Frey, Moritz Passenberg, Johannes Korth, Jaqueline Zmudzinski, Olympia E. Anastasiou, Fuat H. Saner, Michael Jahn, Christian M. Lange, Katharina Willuweit

**Affiliations:** 1 Department of Gastroenterology and Hepatology, University Hospital Essen, University of Duisburg-Essen, Hufelandstr. 55, 45147 Essen, Germany; jassin.rashidi@uk-essen.de (J.R.-A.); alexandra.frey@uk-essen.de (A.F.); moritz.passenberg@uk-essen.de (M.P.); jaqueline.zmudzinski@uk-essen.de (J.Z.); christian.lange@uk-essen.de (C.M.L.); 2 Department of Nephrology, University Hospital Essen, University of Duisburg-Essen, Hufelandstr. 55, 45147 Essen, Germany; johannes.korth@uk-essen.de (J.K.); michael.jahn@uk-essen.de (M.J.); 3 Institute for Virology, University Hospital Essen, University of Duisburg-Essen, Virchowstr. 179, 45147 Essen, Germany; olympia.anastasiou@uk-essen.de; 4 Department of General, Visceral and Transplantation Surgery, University Hospital Essen, University of Duisburg-Essen, Hufelandstr. 55, 45147 Essen, Germany; fuat.saner@uk-essen.de

**Keywords:** SARS-Cov-2, vaccination, liver transplant recipients, liver transplantation, COVID-19

## Abstract

Vaccination against SARS-CoV-2 infection is currently approved and shows favorable outcomes, but little known about antibody responses in solid organ transplant recipients, since these patients are known to have an impaired immune response upon vaccination and have not been included in admission studies. We therefore analyzed immunogenicity in 43 liver transplant (LT) recipients in a median of 15 days (IQR, 12–24) after receiving two doses of the mRNA-based SARS-CoV-2 vaccine BNT162b2 following the standard protocol, and compared these results to a control group consisting of 20 healthcare workers (HCWs). Thirty-four of the 43 (79%) LT recipients developed antibodies, compared to 20 out of 20 (100%) in the control group (*p* = 0.047). The median SARS-CoV-2 IgG titer was significantly lower in the LT recipients compared to the control group (216 vs. >2080 BAU/mL, *p* = 0.0001). Age and sex distribution was similar in the LT patients that developed antibodies after vaccination compared to those who did not. Interestingly, the patients who received mycophenolate mofetil exhibited a reduced vaccination response compared to the other LT patients (5 of 11 (45.5%) vs. 29 of 32 (90.6%), *p* = 0.004). In conclusion, our data reveal lower immunogenicity of SARS-CoV-2 vaccine BNT162b2 in LT patients compared to the control group, but still show superior results compared to other solid organ transplant recipients reported so far.

## 1. Introduction

The novel severe acute respiratory syndrome coronavirus-2 (SARS-CoV-2), the cause of coronavirus disease 2019 (COVID-19), is a serious global health hazard [1], and mortality rates of up to 20% have been reported in solid organ transplant (SOT) recipients [2,3,4]. New messenger RNA (mRNA)-based SARS-CoV-2 vaccines have been evaluated and found to have efficacy of 94–95% in preventing COVID-19-associated illness in phase 3 placebo-controlled trials [5,6]. Regarding SOT recipients, there is limited information about the efficacy and risks of vaccination, since this group of patients has not been included in admission studies, and SOT recipients are known to respond unsatisfactorily upon vaccination with different vaccines [7,8,9]. Still, vaccination of SOT patients is prioritized in many countries, and regarding liver transplant (LT) recipients, vaccination is recommended by professional international societies [10,11]. German guidelines note that LT recipients can receive the SARS-CoV-2 vaccination, and those patients with a high risk of suffering from severe COVID-19 disease, such as those of older age or with relevant comorbidities, should be prioritized [12]. So far, different studies have been published regarding SARS-CoV-2 vaccination in SOT recipients with mostly low, but partly acceptable, immunogenicity [13,14,15,16]. Still, data regarding the vaccination of SOT patients is scarce, and further studies are necessary. 

This study analyzed the immune response of 43 LT recipients after vaccination with two doses of the mRNA-based SARS-CoV-2 vaccine BNT162b2 (Pfizer-BioNTech, Pfizer Inc., New York City, NY, USA, and BioNTech SE, Mainz, Germany) in February and March 2021. The results were compared to the antibody response of 20 healthcare workers (HCWs) that were also vaccinated using the same protocol. 

## 2. Materials and Methods

Forty-three LT recipients were vaccinated with the mRNA-based SARS-CoV-2 vaccine BNT162b2 (Pfizer-BioNTech, Pfizer Inc., New York City, NY, USA, and BioNTech SE, Mainz, Germany) according to the standard protocol with a median time of 26 days (IQR 21–37) between the first and second vaccination. LT recipients aged under 18 and pregnant LT recipients were excluded from the study. In a median of 15 days (IQR, 12–24) after the second intramuscular vaccination, serum samples were tested for SARS-CoV-2 IgG against the Spike glycoprotein using an approved anti-SARS-CoV-2 IgG CLIA (LIAISON^®^ SARS-CoV-2 TrimericS IgG assay, Diasorin, Saluggia, Italy). An arbitrary units per milliliter (AU/mL) ratio of <13.0 was considered to be negative and of ≥13.0 to be positive, according to the manufacturer’s recommendations. A conversion of arbitrary units per milliliter to binding antibody units (BAU/mL) that correlate with the WHO standard is possible using the following equation: 2.6* AU/mL = BAU/mL, with 800.0 AU/mL (2080 BAU/mL) being the upper limit of quantification without dilution of the CLIA.

In addition, the antibody response of both the LT patients and the HCWs was compared after two intramuscular vaccinations at the University Hospital Essen in January 2020, using the same vaccination, sampling, and testing protocol in both groups. Quantitative variables are reported as median (IQR). Fisher’s exact test and Mann–Whitney *U*-test were used to compare the results between groups. A *p*-value of ≤0.05 was considered statistically significant. Statistical analysis was performed with SPSS 27 statistical software (IBM SPSS Statistics; IBM Corporation, Chicago, IL, USA), and GraphPad Prism version 8 for windows (GraphPad Software, San Diego, CA, USA) was used for illustration. This study was conducted according to the guidelines of the Declaration of Helsinki and approved by the ethics committee of the Medical Faculty of the University Duisburg-Essen (20-9753-BO).

## 3. Results

### 3.1. Baseline Characteristics

In total, 43 LT recipients followed up by our outpatient clinic were enrolled. Out of these, 26 (60.5%) were male and 17 (39.5%) were female (Table 1). Most frequent indications for LT were hepatocellular carcinoma (*n* = 10, 23%) and primary sclerosing cholangitis (*n* = 7, 16%), followed by alcohol-induced liver cirrhosis (*n* = 6, 14%), hepatitis C virus-induced liver cirrhosis, and acute liver failure and Wilson’s disease (each *n* = 3, 7%) (Table 2). The median time from LT to first vaccination was eight years (IQR, 4–12) and the median age at first vaccination was 57 years (IQR, 49–64). The median time between the first and second vaccinations was 26 days (IQR, 21–37), and the median time between the second vaccination and SARS-CoV-2 antibody detection was 15 days (IQR, 12–24). The demographic data are depicted in more detail in Table 1. Regarding immunosuppressive therapy at the time of vaccination, most patients were receiving tacrolimus-based immunosuppression (*n* = 40, 93%). Out of these, most patients were receiving a combination therapy of tacrolimus and everolimus (*n* = 22, 55%) or of tacrolimus and mycophenolate mofetil (MMF) (*n* = 11, 28%). Seven patients (18%) were receiving tacrolimus monotherapy, while two patients (5%) obtained monotherapy with cyclosporine A and one patient (2%) monotherapy with everolimus (Table 3). Two (5%) of our patients were receiving additional immunosuppression with low-dose corticosteroids at the time of enrolment, and none received high-dose prednisolone in the 12 months prior to vaccination. 

Regarding the group of HCWs, nine (45%) were male and 11 (55%) were female. The median age at first vaccination was 43.5 years (IQR, 38–53.5). The median time between the first and second vaccinations was 22 days, and the median time between the second vaccination and SARS-CoV-2 antibody detection was 13 days. 

The LT recipients were significantly older than the control group (57 vs. 43.5 years, *p* = 0.002), and the time between the second vaccination and antibody measurement was slightly longer for the LT group (15 vs. 13 days, *p* = 0.033). None of the patients or HCWs had a history of SARS-CoV-2 infection before vaccination. 

### 3.2. Antibody Response and Titer after SARS-CoV-2 Vaccination

While all HCWs (100%) developed antibodies after SARS-CoV-2 vaccination, only 34 of the 43 LT recipients (79%) showed an antibody response with the detection of SARS-CoV-2 IgG and nine patients (21%) remained negative (*p* = 0.047) (Table 1). Out of these, six were male and three were female, with a median age of 59 years (IQR, 53.5–64.5) at first vaccination. The median time between the second vaccination and antibody detection was 15 days (IQR 13-25). All of these patients received tacrolimus-based combinations of immunosuppression (combined with everolimus in three patients and with MMF in six patients).

**Figure 1:**
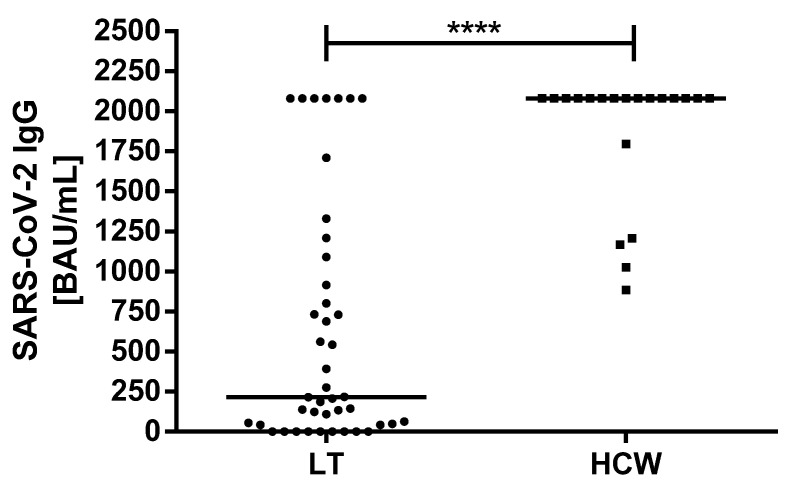
Comparison of the binding antibody units per milliliter (BAU/mL) ratio of SARS-CoV-2 IgG antibodies of the LT recipients and HCWs after the second vaccination. HCW: healthcare worker; BAU: binding antibody units; ml: milliliter; SARS-CoV-2: severe acute respiratory syndrome coronavirus type 2. **** *p* < 0.0001.

The median antibody titer was significantly lower in LT patients compared to the control group (216 vs. >2080, *p* = 0.0001) (Figure 1). Even when taking into account only seropositive LT recipients, the median SARS-CoV-2 IgG titer was still significantly lower compared to the control group (552.7 vs. >2080 BAU/mL) (*p* = 0.0001). No episodes of acute cellular rejection were observed after vaccination (Table 1). 

When comparing the groups of seropositive and seronegative LT recipients, no significant differences were observed with respect to sex (*p* = 1.00), age at LT (*p* = 0.97), age at first vaccination (*p* = 0.35), time between LT and first vaccination (*p* = 0.10), time between first and second vaccinations (*p* = 0.55), or time between second vaccination and antibody detection (*p* = 0.85) (Table 4).

Regarding immunosuppressive therapy, patients receiving MMF developed less frequently antibodies compared to patients who were not taking MMF (*p* = 0.004). Receiving tacrolimus (*p* = 1.00) or everolimus (*p* = 0.26) had no statistically significant impact on antibody detection. 

## 4. Discussion

We herein described the immunogenicity data of 43 LT recipients after standard protocol-based vaccination of two doses of the mRNA-based SARS-CoV-2 vaccine BNT162b2 (Pfizer-BioNTech, Pfizer Inc., New York City, NY, USA, and BioNTech SE, Mainz, Germany) in comparison to a healthy control group. Although immunogenicity was significantly lower in the LT patients than in our control group, it still showed acceptable results, indicating the potential to provide humoral immunity in this group of patients. 

Large phase 3 trials showed an efficacy of 95% by the mRNA-based vaccine BNT162b2 (Pfizer-BioNTech, Pfizer Inc., New York City, NY, USA, and BioNTech SE, Mainz, Germany) [6]. Immunocompromised patients after SOT are known to have an impaired immune response to vaccinations [7,8], and since SOT recipients were not included in the mentioned trials, little is known about immunogenicity in this patient collective. 

Regarding SOTs other than LT, poor immunogenicity has been described in initial results reporting response rates of 22–36.4% for kidney transplant recipients [13,16]. A different study analyzing an assorted group of both kidney and heart transplant recipients demonstrated a response rate of 58.8% [14]. 

To the best of our knowledge, there is only one recently published study regarding a homogenous group of LT recipients by Rabinowich et al., describing an antibody response rate of 47.5% 10–20 days after the second vaccination with BNT162b2, thereby showing inferior immunogenicity compared to our cohort (79%). The serum IgG titer was significantly lower in the group of seropositive LT recipients compared to their control group of healthy volunteers, as was the case in our cohort. 

The liver is known to show higher immune tolerance after transplantation, leading to lower dosage of immunosuppressive medication compared to other SOT recipients in general [17,18], which can explain the poor results regarding immunogenicity after kidney transplantation and the superior results in LT recipients. However, in comparison to the data of Rabinowich et al., our cohort showed favorable outcomes with a higher rate of antibody response. Of course, sample size might be a possible reason for this difference, but still, there are other factors that could provide plausible explanations. Among others, an older age and high-dosage treatment with steroids were described by Rabinowich et al. as predictors of the absence of an antibody response, and older age was also reported to be a risk factor in a previous study regarding assorted SOT recipients after the application of the first vaccination dose by Boyarsky et al. [19]. The median age was somewhat lower in our cohort compared to the mean age of the LT recipients in the study of Rabinowich et al.; however, we can only speculate about the significance of this difference. Although there was no significant difference regarding age in the groups showing positive and negative antibody reactions in our cohort, it has to be considered that our cohort was small, and age can still be an important factor. The sex distribution was slightly different as well, with more female patients in our cohort (39.5% vs. 30%), which might be another reason for the observed differences in both studies, since women tend to develop higher antibody responses following vaccination [20]. 

Additionally, 30% of the patients of Rabinowich et al. received medication with prednisolone, and 20% had been treated with high-dose steroids within the last year prior to vaccination, whereas only 5% of the patients in our cohort were treated with steroids, and none received high-dose steroids in the year before. Since high-dose prednisolone treatment impaired immunogenicity significantly in the cohort of Rabinowich et al., corticosteroids might be an additional factor explaining the differences in both LT cohorts. 

Regarding immunosuppressive therapy other than prednisolone, patients receiving MMF showed inferior immunogenicity, which is consistent with previous works in SOT recipients [14,15,16]. There are single descriptions of episodes with acute cellular rejection after SARS-CoV-2 vaccination in kidney [21] as well as liver transplant patients [22]. However, no rejection episodes were observed in our cohort. 

Our study has limitations, most importantly the small sample size. Due to the non-normal distribution of the data, we used the Mann–Whitney *U*-test for data analysis and group comparisons, which one should keep in mind when interpreting the results. Furthermore, additional characterization of the immune response, such as analyzing memory B and T cell-mediated immune response or neutralizing the capacity of antibodies, could be of interest and were not considered in this study. In addition, antibody detection was undertaken relatively early after completion of the vaccination, with a median of 15 days between the second dose and antibody detection, although it is known that antibody production gets stronger with time [5]. For these reasons, further clinical trials are of need, and long-term results regarding the antibody response upon vaccination will be of crucial interest. 

## 5. Conclusions

Our data showed lower immunogenicity in a group of LT recipients compared to a healthy control group, but still provide considerably superior results in comparison to previous studies regarding LT and non-LT SOT recipients. In addition, immunogenicity was significantly inferior in patients receiving MMF as immunosuppressive medication compared to patients who were not receiving MMF. 

These findings allow a cautiously optimistic outlook on the outcomes of SARS-CoV-2 vaccination in LT patients. These results have to yet be confirmed in larger cohorts and the evaluation of longitudinal vaccine responses, and efficacy of immune responses against morbidity and mortality will be of crucial importance. Still, limited immunogenicity and possible immunization failure should always be kept in mind in patients receiving SARS-CoV-2 vaccination, emphasizing the need for monitoring the immune response, as well as maintaining personal precautions in social interactions until further investigations of the abovementioned issues.

## Figures and Tables

**Table 1:** Patient characteristics are presented as absolute number *n* and percentage or median and interquartile range.

Patient Characteristics	LT Recipients *n*/(%)	HCWs *n*/(%)	*p*-Value
Total patient number	43	20	-
Sex (male/female)	26(60.5)/17 (39.5)	9 (45)/11 (55)	0.286
SARS-CoV-2 IgG detectability rate	34 (79)	20 (100)	0.047
	**Median (IQR)**	**Median (IQR)**	
Age of recipient at LT [years]	47 (36–54)	-	-
Time between LT and first dose [years]	8 (4–12)	-	-
Age at first dose [years]	57 (49–64)	43.5 (38–53.5)	0.002
Time between first and second doses [days]	26 (21–37)	22	0.448
Time between second dose and SARS-CoV-2 Ab detection [days]	15 (12–24)	13	0.033
SARS-CoV-2 IgG (BAU/mL)	216 (43.4–1090)	>2080 (1865.5 to >2080)	0.0001

LT: liver transplantation; HCWs: healthcare workers; Ab: antibody; BAU: binding antibody units; IQR: interquartile range.

**Table 2:** The indication for liver transplantation is shown as absolute number *n* and percentage.

Indication for LT	*n* (%)
Hepatocellular carcinoma	10 (23)
Primary sclerosing cholangitis	7 (16)
Alcohol-induced liver cirrhosis	6 (14)
Hepatitis C virus-induced liver cirrhosis	3 (7)
Acute liver failure	3 (7)
Wilson’s disease	3 (7)
Cryptogenic liver cirrhosis	2 (4.7)
α-1 antitrypsin deficiency	2 (4.7)
Others ^1^	7 (16)

^1^ Others: autoimmunhepatitis, polycystic liver and kidney disease, hepatitis B virus-induced liver cirrhosis, Alagille syndrome, and neuroendocrine tumor (*n* = 1 each).

**Table 3:** The immunosuppressive therapy is given as absolute number *n* and percentage.

Immunosuppressive Therapy	*n* (%)
Tacrolimus-based	40 (93)
Tacrolimus + everolimus	22 (55)
Tacrolimus + MMF	11 (28)
Tacrolimus monotherapy	7 (18)
Cyclosporine A	2 (5)
Everolimus	1 (2)

MMF: mycophenolate mofetil.

**Table 4:** Comparison of the LT recipients with negative and positive SARS-CoV-2 serology after vaccination. Patient characteristics are presented as absolute number *n* and percentage or median and interquartile range.

Patient Characteristics	LT Recipients SARS-CoV-2 IgG Positive *n*/(%)	LT Recipients SARS-CoV-2 IgG Negative *n*/(%)	*p*-Value
Total patient number	34 (79)	9 (21)	-
Sex (male/female)	20 (59)/14 (41)	6 (67)/3 (33)	1.00
**Immunosuppression**			
Tacrolimus (*n* = 40)	31 (77.5)	9 (22.5)	1.00
No Tacrolimus (*n* = 3)	3 (100)	-	
Everolimus (*n* = 23)	20 (87)	3 (13)	0.26
No Everolimus (*n* = 20)	14 (70)	6 (30)	
MMF (*n* = 11)	5 (45.5)	6 (54.5)	0.004
No MMF (*n* = 32)	29 (90.6)	3 (9.4)	
	**Median (IQR)**	**Median (IQR)**	
Age of recipient at LT [years]	48.5 (36–54)	45 (40–55)	0.97
Time between LT and first dose [years]	6.5 (3.7-12)	12 (5.5-14.5)	0.10
Age at first dose [years]	56.5 (46–64)	59 (53.5-64.5)	0.35
Time between first and second doses [days]	28 (21–37)	21 (21–40)	0.55
Time between second dose and SARS-CoV-2 Ab detection [days]	14 (12–25)	15 (13–25)	0.85
SARS-CoV-2 IgG (BAU/mL)	552.7 (137.3-1425)	-	-

LT: liver transplantation; HCW: healthcare workers; Ab: antibody; BAU: binding antibody units; IQR: interquartile range.

## Data Availability

The data that support the findings of this study are available from the corresponding author upon reasonable request.
